# *De Novo* Origin of Protein-Coding Genes in Murine Rodents

**DOI:** 10.1371/journal.pone.0048650

**Published:** 2012-11-21

**Authors:** Daniel N. Murphy, Aoife McLysaght

**Affiliations:** Smurfit Institute of Genetics, University of Dublin, Trinity College, Dublin, Ireland; Hebrew University at Jerusalem, The Alexander Silberman Institute of Life Sciences, Israel

## Abstract

**Background:**

New genes in eukaryotes are created through a variety of different mechanisms. *De novo* origin from non-coding DNA is a mechanism that has recently gained attention. So far, *de novo* genes have been described in a handful of organisms, with *Drosophila* being the most extensively studied. We searched for genes that have appeared *de novo* in the mouse and rat lineages.

**Methodology:**

Using a rigorous and conservative approach we identify 75 murine genes (69 mouse genes and 6 rat genes) for which there is good evidence of *de novo* origin since the divergence of mouse and rat. Each of these genes is only found in either the mouse or rat lineages, with no candidate orthologs nor evidence for potentially-unannotated orthologs in the other lineage. The veracity of each of these genes is supported by expression evidence. Additionally, their presence in one lineage and absence in the other cannot be explained by sequencing gaps. For 11 of the 75 candidate novel genes we could identify a mouse-specific mutation that led to the creation of the open reading frame (ORF) specifically in mouse. None of the six rat-specific genes had an unequivocal rat-specific mutation creating the ORF, which may at least be partly due to lower data quality for that genome.

**Conclusions:**

All 75 candidate genes presented in this study are relatively small and encode short peptides. A large number of them (51 out of 69 mouse genes and 3 out of 6 rat genes) also overlap with other genes, either within introns, or on the opposite strand. These characteristics have previously been documented for *de novo* genes. The description of these genes opens up the opportunity to integrate this evolutionary analysis with the rich experimental data available for these two model organisms.

## Introduction

The origin of a new gene can occur through several mechanisms such as duplication, exon shuffling, and the fusion or fission of existing genes [1]. The characteristic feature of these mechanisms is a pre-existing parent gene, which, in whole or in part, gives rise to the new gene. A classic example of genes arising partly though duplication, and partly through *de novo* mechanisms, is the evolution of the antifreeze glycoprotein in Arctic cod and in Antarctic notothenioid fish [2]. Another possible mechanism, but one that is rarely observed, is the creation of completely novel genes from previously non-coding DNA. So far, evidence for the creation of protein-coding *de novo* genes has only been described in a small group of eukaryotes consisting of yeast [3], [4], Drosophila [5], [6], [7], [8], [9], [10], the protozoan *Plasmodium vivax* [11], ancestral primates [12], human [13], [14], [15], [16], and rice [17]. A *de novo* gene has also been discovered in mouse, though it does not encode a protein and is instead thought to produce a non-coding RNA [18].

A large fraction of the open reading frames (ORFs) in mammalian genomes is suspected to be functionally meaningless, as they show no evidence of evolutionary conservation with other species. However, this is not sufficient evidence to discount the possibility that these ORFs do in fact encode functioning proteins. By definition, *de novo* genes are unique to a specific lineage, and as such may be responsible, or partly responsible, for phenotypes that set one species apart from its closest relatives [19]. However, due to their exclusive presence in one lineage or species, these genes are less likely to have been the subject of functional analyses.

We searched for *de novo* genes that have appeared in the mouse and rat lineages since their divergence 14–40 million years ago [20], [21], [22], [23]. The practical uses of having a list of known *de novo* genes in mouse and rat are plentiful, and the two species provide researchers with platforms upon which such genes can be studied, something that is lacking for human-specific cases. In particular, rodent genes can be easily subjected to functional analyses such as knockout studies.

For this study we used rigorous and conservative criteria to ensure the exclusion of artefacts such as sequencing and annotation errors, ultimately ending up with a rather small, but well-supported, list of candidates.

## Results

### Identification of mouse and rat genes with no protein-coding matches

**Figure 1:**
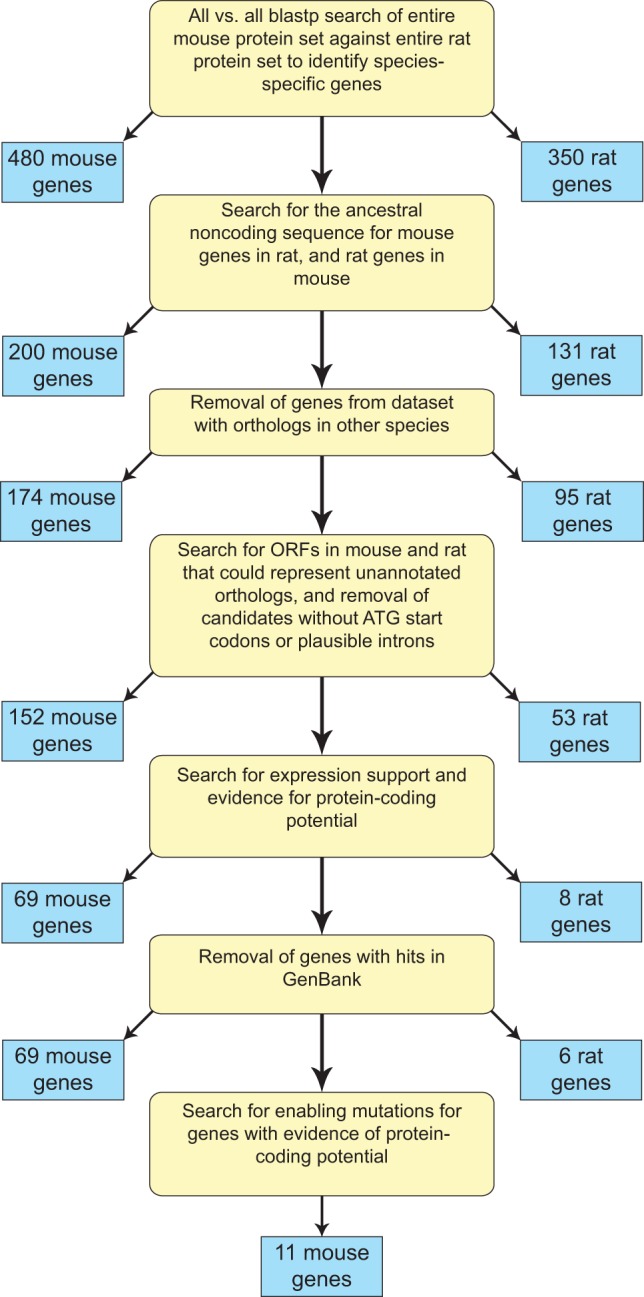
Flowchart summary of methods used. Each of the steps taken to obtain the sets of mouse and rat *de novo* genes is shown in yellow boxes. The numbers of mouse and rat genes remaining after each step are shown in blue boxes.

We initially compared the complete set of protein coding genes from mouse and rat using blastp to determine all genes found in one species and not the other, thereby obtaining a preliminary list of 480 and 350 candidate novel genes in mouse and rat, respectively. We then excluded genes with plausible orthologs in any other species, as these may be explained by lineage-specific loss (Fig. 1).

We considered the possibility that genuine, but unannotated, orthologs might exist in the other rodent genome. We searched the rat genome for sequences homologous to each of the mouse genes, and the mouse genome for sequences homologous to each of the rat genes. If the corresponding homologous sequence was not identifiable then the gene was removed from the list of candidates as we cannot exclude the possibility that the gene is present but unsequenced. Once the homologous sequence was identified we examined it for evidence of protein-coding capacity (i.e., an unannotated, but plausible ortholog). All potential ORFs were translated into protein sequences, and these were compared to the proteins encoded by the candidate *de novo* gene in question. Cases where a potential ORF aligned to at least 50% of the candidate novel gene with at least 60% identity were discarded. After completion of these rigorous quality control steps 152 and 53 candidate *de novo* genes remained for mouse and rat, respectively.

### Evidence for transcription and protein-coding potential of the *de novo* genes

Evidence that a *de novo* gene is expressed and translated into a protein is significant in arguing for its authenticity. In previous studies of entirely *de novo* genes only one gene in yeast and three in human had some high throughput mass spectrometry support for their protein-coding potential [3], [13]. We searched microarray and EST databases and found evidence of transcription for 69 candidate novel mouse genes and 6 rat genes (Table 1 and Table 2, respectively).

**Table 1:** Summary of the 69 candidate mouse novel genes.

EnsEMBL ID	Genomic location	Length (aa)	Overlapping genes	Number of exons^a^	Knockouts	Peptide evidence^b^	Expression evidence^c^
ENSMUSG00000075472*	11:106683070..106683258:-1	62	ENSMUSG00000018363	1		PeptideAtlas (3)	ArrayExpress, Genevestigator
ENSMUSG00000078251	11:113331568..113331885:1	106	ENSMUSG00000041654	1		PeptideAtlas (2)	ArrayExpress, Bgee, Genevestigator
ENSMUSG00000074740*	19:60865923..60867569:1	84	ENSMUSG00000024991	1	knocked out in cell line, no phenotype in live mouse yet	PRIDE (1), PeptideAtlas (4)	Bgee, Genevestigator
ENSMUSG00000066371*	9:107774792..107775570:1	129	ENSMUSG00000032582	1	knocked out in cell line, no phenotype in live mouse yet	PRIDE (3), PeptideAtlas (4)	ArrayExpress, Bgee, Genevestigator
ENSMUSG00000051562*	9:122929985..122930362:-1	125		1		PRIDE (4), PeptideAtlas (5)	ArrayExpress, Bgee, Genevestigator
ENSMUSG00000072684	14:26484519..26486217:1	121	ENSMUSG00000007817	1	knocked out in cell line, no phenotype in live mouse yet	PRIDE (3), PeptideAtlas (7)	Bgee, Genevestigator
ENSMUSG00000075582*	14:57719305..57719652:1	115	ENSMUSG00000046352	1		PRIDE (1), PeptideAtlas (4)	Genevestigator
ENSMUSG00000056640*	14:70057982..70058305:-1	107	ENSMUSG00000085092, ENSMUSG00000034205	1		PRIDE (2), PeptideAtlas (6)	Genevestigator
ENSMUSG00000054990*	18:25288507..25288980:-1	157	ENSMUSG00000034295, ENSMUSG00000024269	1		PRIDE (2), PeptideAtlas (3)	ArrayExpress, Genevestigator
ENSMUSG00000074880*	10:80257231..80258205:1	115	ENSMUSG00000061589	1		PeptideAtlas (3)	Genevestigator
ENSMUSG00000055108	10:98588633..98588830:-1	65	ENSMUSG00000019952	1		PRIDE (1), PeptideAtlas (3)	ArrayExpress, Bgee, CleanEx, Genevestigator, GermOnline
ENSMUSG00000074246*	8:74148472..74148777:-1	101	ENSMUSG00000034807	1		PRIDE (2), PeptideAtlas (6)	Bgee, Genevestigator
ENSMUSG00000072431*	8:80040955..80042477:1	122	ENSMUSG00000037148	1		PeptideAtlas (5)	
ENSMUSG00000037982	8:83537426..83539566:1	164	ENSMUSG00000038250	1	knocked out in cell line, no phenotype in live mouse yet	PRIDE (3), PeptideAtlas (10)	ArrayExpress, Bgee, Genevestigator
ENSMUSG00000078283*	6:91712358..91712738:-1	126	ENSMUSG00000030098	1		PRIDE (4), PeptideAtlas (5)	ArrayExpress, Bgee, Genevestigator
ENSMUSG00000079446*	6:100654749..100657668:1	108	ENSMUSG00000030074	2 (1)		PeptideAtlas (6)	Genevestigator
ENSMUSG00000073546	18:65466084..65469530:1	103	ENSMUSG00000032845	2 (1)		PRIDE (3), PeptideAtlas (5)	ArrayExpress, Genevestigator
ENSMUSG00000072655*	6:149234588..149234908:-1	106		1		PeptideAtlas (8)	Genevestigator
ENSMUSG00000063757	7:4985680..4988370:1	138	ENSMUSG00000043290	1		PeptideAtlas (5)	ArrayExpress, Bgee, Genevestigator
ENSMUSG00000078384	7:28886093..28886566:-1	157	ENSMUSG00000047730	1		PeptideAtlas (3)	Genevestigator
ENSMUSG00000070574	7:51932335..51933605:1	172		2 (2)		PRIDE (4), PeptideAtlas (16)	ArrayExpress, Bgee, Genevestigator
ENSMUSG00000074118	7:53038302..53039837:-1	106	ENSMUSG00000062044	2 (2)		PRIDE (1)	Genevestigator
ENSMUSG00000074087*	7:66486831..66487037:-1	68	ENSMUSG00000025326	1		PRIDE (2), PeptideAtlas (3)	Genevestigator
ENSMUSG00000073994*	7:107610439..107610750:-1	103	ENSMUSG00000047248	1		PeptideAtlas (5)	Bgee, Genevestigator
ENSMUSG00000044407	17:10512525..10513094:-1	189		1	knockout and mutations in other databases cause phenotypes including mortality	PRIDE (3), PeptideAtlas (10)	Bgee, Genevestigator, Eurexpress
ENSMUSG00000073464*	17:11924400..11924819:-1	139	ENSMUSG00000023826	1		PRIDE (3), PeptideAtlas (4)	Genevestigator
ENSMUSG00000049740	7:114768047..114768379:-1	110	ENSMUSG00000036528	1		PeptideAtlas (3)	ArrayExpress, Bgee, Genevestigator
ENSMUSG00000067798	5:20045621..20046234:1	129	ENSMUSG00000040003	2 (2)	several phenotypes including mortality	PRIDE (1), PeptideAtlas (4)	Bgee, Genevestigator
ENSMUSG00000078181*	5:31947374..31948728:1	110	ENSMUSG00000029142, ENSMUSG00000029136	1		PeptideAtlas (8)	Bgee, Genevestigator
ENSMUSG00000072962	5:44491681..44493752:1	153	ENSMUSG00000029086	1		PRIDE (5), PeptideAtlas (12)	Bgee, Genevestigator
ENSMUSG00000057354	5:115886084..115886548:-1	154	ENSMUSG00000054256	1		PRIDE (4), PeptideAtlas (8)	ArrayExpress, Bgee, Genevestigator
ENSMUSG00000072639*	5:122689186..122689530:-1	114	ENSMUSG00000064267	1		PRIDE (1), PeptideAtlas (4)	Bgee, Genevestigator
ENSMUSG00000063155*	5:130698013..130698477:-1	154	ENSMUSG00000053094	1		PeptideAtlas (3)	ArrayExpress, Bgee, Genevestigator
ENSMUSG00000021206	5:139853651..139856007:1	139	ENSMUSG00000053553, ENSMUSG00000044197	1		PRIDE (4), PeptideAtlas (7)	ArrayExpress, Bgee, Genevestigator
ENSMUSG00000073875	4:42090151..42090576:-1	141		1		PeptideAtlas (1)	
ENSMUSG00000070700*	4:133559278..133560802:1	120	ENSMUSG00000050966	1		PRIDE (1), PeptideAtlas (6)	ArrayExpress, Bgee, Genevestigator
ENSMUSG00000053280*	17:31570894..31571349:-1	151	ENSMUSG00000041119	1		PRIDE (2), PeptideAtlas (4)	ArrayExpress, Genevestigator
ENSMUSG00000066178	4:136018165..136019892:1	148		1	knocked out in cell line, no phenotype in live mouse yet	PeptideAtlas (5)	ArrayExpress, Bgee, Genevestigator
ENSMUSG00000073719	4:144562623..144563060:-1	145	ENSMUSG00000020220	1	knocked out in cell line, no phenotype in live mouse yet	PeptideAtlas (6)	ArrayExpress, Genevestigator
ENSMUSG00000054354*	17:34118437..34122296:1	113		1		PRIDE (1), PeptideAtlas (1)	ArrayExpress, Bgee, Genevestigator
ENSMUSG00000069012*	3:54517759..54517881:-1	40	ENSMUSG00000027751	1		PeptideAtlas (2)	Genevestigator
ENSMUSG00000074517	3:82931484..82932008:-1	174		1		PRIDE (3), PeptideAtlas (4)	ArrayExpress, Bgee, Genevestigator
ENSMUSG00000074318*	3:107690458..107690682:-1	74	ENSMUSG00000040600	1		PeptideAtlas (4)	Bgee, Genevestigator
ENSMUSG00000074237	3:127741612..127743579:1	127		1		PRIDE (2), PeptideAtlas (4)	Genevestigator
ENSMUSG00000054773	3:156871295..156871534:-1	79	ENSMUSG00000040037	1		PeptideAtlas (2)	ArrayExpress, Bgee, Genevestigator
ENSMUSG00000049276*	X:12605226..12605390:-1	54		1		PRIDE (1), PeptideAtlas (2)	ArrayExpress, Bgee, Genevestigator
ENSMUSG00000073231	X:39880439..39881044:1	80	ENSMUSG00000016150	1		PeptideAtlas (2)	ArrayExpress, Bgee, Genevestigator
ENSMUSG00000072960	X:133276321..133277682:1	141	ENSMUSG00000031422, ENSMUSG00000087368	1	knocked out in cell line, no phenotype in live mouse yet	PRIDE (2), PeptideAtlas (4)	ArrayExpress, Genevestigator
ENSMUSG00000072913*	X:147275576..147275953:-1	125	ENSMUSG00000087149	1		PeptideAtlas (12)	Genevestigator
ENSMUSG00000069875	2:4088273..4088632:1	119	ENSMUSG00000026657	1	knocked out in cell line, no phenotype in live mouse yet	PRIDE (2), PeptideAtlas (14)	Genevestigator
ENSMUSG00000073388	17:47026744..47027154:-1	136		1	knocked out in cell line, no phenotype in live mouse yet	PRIDE (1), PeptideAtlas (6)	ArrayExpress, Bgee, Genevestigator
ENSMUSG00000074989*	2:104836539..104836859:-1	106	ENSMUSG00000045106	1		PeptideAtlas (3)	Bgee, Genevestigator
ENSMUSG00000074940*	2:112201851..112202165:-1	104	ENSMUSG00000027130	1		PRIDE (2), PeptideAtlas (12)	ArrayExpress, Genevestigator
ENSMUSG00000044744	1:33726688..33727557:1	184		1		PRIDE (2), PeptideAtlas (7)	ArrayExpress, Bgee, Genevestigator
ENSMUSG00000080025*	1:37474621..37474944:-1	107	ENSMUSG00000026112	1		PeptideAtlas (3)	ArrayExpress, Genevestigator
ENSMUSG00000073694*	1:46232431..46232727:1	98		1	knocked out in cell line, no phenotype in live mouse yet	PRIDE (2), PeptideAtlas (5)	Genevestigator
ENSMUSG00000073531*	1:158973680..158974325:1	71		1		PeptideAtlas (4)	Bgee, Genevestigator
ENSMUSG00000054546*	15:27505467..27505868:-1	133	ENSMUSG00000022265	1		PRIDE (1), PeptideAtlas (4)	ArrayExpress, Bgee, Genevestigator
ENSMUSG00000078299*	15:64119151..64119294:-1	47		1		PeptideAtlas (2)	ArrayExpress, Bgee, Genevestigator
ENSMUSG00000078298*	15:64160033..64160371:-1	112		1		PeptideAtlas (4)	Genevestigator
ENSMUSG00000018006*	15:78501215..78506545:1	158	ENSMUSG00000043460	4 (4)		PeptideAtlas (4)	ArrayExpress, Bgee, Genevestigator
ENSMUSG00000075433*	15:97580292..97581586:1	169	ENSMUSG00000022469	1		PeptideAtlas (8)	Bgee, Genevestigator
ENSMUSG00000043805*	15:102888711..102889043:-1	111		1		PeptideAtlas (1)	Genevestigator
ENSMUSG00000055849*	13:25081073..25081390:-1	105	ENSMUSG00000021340	1		PRIDE (1), PeptideAtlas (3)	ArrayExpress, Bgee, Genevestigator
ENSMUSG00000047061	13:45173740..45174554:1	144	ENSMUSG00000078915	1		PeptideAtlas (12)	ArrayExpress, Bgee, Genevestigator
ENSMUSG00000051555*	13:55723728..55724168:-1	146		1	knocked out in cell line, no phenotype in live mouse yet	PeptideAtlas (5)	Genevestigator
ENSMUSG00000048603	13:99086377..99087259:1	124	ENSMUSG00000021660	1	knocked out in cell line, no phenotype in live mouse yet	PRIDE (4), PeptideAtlas (6)	ArrayExpress, Bgee, Genevestigator
ENSMUSG00000053556*	12:81291858..81292307:1	149	ENSMUSG00000015143	1		PeptideAtlas (8)	ArrayExpress, Genevestigator
ENSMUSG00000084085	11:18909131..18911186:1	122	ENSMUSG00000020160	2 (2)		PRIDE (1), PeptideAtlas (5)	Genevestigator

a – If the number of exons is greater than 1, the number of exons in which the coding sequence is contained is shown in brackets.

b – Peptide evidence is shown with the databases in which the peptides are found followed by the number of unique peptides.

c – Databases are shown that contain the expression evidence, in the form of EST and microarray data, for each of the respective genes.

* Retired in EnsEMBL version 61.

**Table 2:** Summary of the 6 candidate rat novel genes.

EnsEMBL ID	Genomic location	length (aa)	Overlapping genes	Number of exons*	Expression evidence
ENSRNOG00000038369	X:68776250..68790582:1	208		4 (4)	Genevestigator
ENSRNOG00000028932	4:80911025..80914836:1	97	Intronic sequence of ENSRNOG00000008063 on opposite strand	2 (2)	Genevestigator
ENSRNOG00000030156	18:18805826..18808748:-1	135		3 (3)	Genevestigator
ENSRNOG00000042175	11:64631466..64632612:1	70		1	Genevestigator
ENSRNOG00000013433	15:47304008..47304328:-1	106	Intronic and exonic sequence of ENSRNOG00000013441 on opposite strand	1	ArrayExpress, Genevestigator, GermOnline
ENSRNOG00000029808	15:60840404..60841246:-1	125	5′ UTR, some intronic and coding sequence of ENSRNOG00000012594 on opposite strand	2 (2)	ArrayExpress, Genevestigator

* If the number of exons is greater than 1, the number of exons in which the coding sequence is contained is shown in brackets.

Expression databases may contain some false positives [24], so to add support for these genes we searched for sequenced peptides in the PeptideAtlas [25] and PRIDE [26] databases. We found no peptide support for any of the rat genes, which is not surprising given that PeptideAtlas contains no rat peptides and PRIDE has very few. We identified uniquely-matching sequenced peptides for 69 mouse genes. Of these, all but three are supported by more than one unique peptide (Table 1).

### Mouse-specific mutations affording protein-coding potential

Apart from presenting a clearer picture of the events that could lead to non-coding sequence becoming an ORF, deciphering the important mutations that facilitated the creation of a *de novo* gene gives further support for its existence. For each of the 69 mouse *de novo* candidates we searched for the orthologous DNA in human and guinea pig using a combination of BLAST and synteny information. These regions in rat had already been determined in a previous step. The orthologous sequences were aligned using MUSCLE [27]. We identified mutations specific to the mouse lineage that resulted in the appearance of an ORF. We termed these mutations “enablers” or “enabling mutations”. The presence of an enabling mutation in mouse that is absent in human, rat and guinea pig is strong evidence for recent lineage-specific creation of the ORF, as the independent inactivation of the gene by an identical mutation in three different lineages is unlikely.

We were able to identify the orthologous sequence in rat, guinea pig and human for only 11 of the 69 candidates (Table 3). For each of the 11 cases we attempted to identify a mouse-specific substitution that created or significantly extended the ORF. In 7 cases the mutations consist of one or two simple indels, while for the other four the transition from non-coding to ORF is less clear and may have involved several independent mutations. Sequence traces for the regions containing the enablers were taken from NCBI (unavailable for guinea pig) in order to ensure there was no ambiguity with regards to the sequence in the relevant enabler regions (Figs. S1, S2, S3, S4, S5, S6, S7, S8, S9, S10, S11).

**Table 3:** Mouse candidates with evidence for transcription, translation and lineage-specific enabler.

EnsEMBL ID	Length (aa)	Peptide evidencea	Expression evidenceb	Enabler in mouse
ENSMUSG00000075472*	62	PeptideAtlas (3)	Gene Expression Atlas, ArrayExpress, Genevestigator	deletion of 5nt causing frameshift
ENSMUSG00000075582*	115	PRIDE (1), PeptideAtlas (4)	Genevestigator	G->A creating start codon
ENSMUSG00000037982	164	PRIDE (3), PeptideAtlas (10)	ArrayExpress, Bgee, Genevestigator, Gene Expression Atlas	T->G removing stop codon
ENSMUSG00000078384	157	PeptideAtlas (3)	Genevestigator	deletion of G resulting in frameshift
ENSMUSG00000057354	154	PRIDE (4), PeptideAtlas (8)	ArrayExpress, Bgee, Genevestigator, Gene Expression Atlas	deletion of G resulting in frameshift
ENSMUSG00000070700*	120	PRIDE (1), PeptideAtlas (6)	ArrayExpress, Bgee, Genevestigator, Gene Expression Atlas	deletion of A resulting in frameshift
ENSMUSG00000074517	174	PRIDE (3), PeptideAtlas (4)	ArrayExpress, Bgee, Genevestigator, Gene Expression Atlas	C->T creating start codon and deletion of C causing frameshift
ENSMUSG00000073388***	136	PRIDE (1), PeptideAtlas (6)	ArrayExpress, Bgee, Genevestigator, Gene Expression Atlas	insertion of 38nt resulting in frameshift and novel protein
ENSMUSG00000075433*	169	PeptideAtlas (8)	Bgee, Genevestigator	T->G creating start codon
ENSMUSG00000043805*	111	PeptideAtlas (1)**	Genevestigator	deletion of C creating start codon, 3 other separate indels causing frameshifts
ENSMUSG00000048603	124	PRIDE (4), PeptideAtlas (6)	ArrayExpress, Bgee, Genevestigator, Gene Expression Atlas	indel of several nt causing a frameshift

a – Peptide evidence is shown with the databases in which the peptides are found followed by the number of unique peptides.

b – Databases are shown that contain the expression evidence, in the form of EST and microarray data, for each of the respective genes.

* Retired in EnsEMBL version 61.

** Only one unique peptide is considered to be weak evidence for the protein-coding potential of the gene.

*** Large ORFs are present in ancestral location in other species but a frameshift means they encode completely different proteins.

These genes are the strongest candidates for having arisen *de novo* as they are completely unique to mouse, they have support in the form of expression and peptide data, and they have unique enablers when compared to the ancestral DNA in other lineages. This all implies that the genes were not present in the mammalian ancestor, and have arisen recently in the mouse lineage.

### What do the genes do?

We searched for any information on the functions of these genes. The International Knockout Mouse Consortium (IKMC) offers a large data repository for mouse knockout data [28] and contains entries for 14 out of the 69 mouse candidates. Twelve of these genes, three of which belong to the most robustly-supported group of 11 *de novo* genes (Table 3; Table 4), have only been knocked out in cell lines so far and have not produced any phenotypes. The remaining two knocked-out genes (ENSMUSG00000067798 and ENSMUSG00000044407) cause morbidity and affect growth, embryogenesis, and the nervous and cardiovascular systems when disrupted. This not only supports the inference that these genes are genuine, but also suggests that they have essential functions. However, for each of these two genes, the knockout covers another gene as well and the phenotypes that are reported may be due to the disruption of the overlapping genes. The gene overlapping with ENSMUSG00000067798 encodes MAGI2, a kinase enzyme involved in several processes and found to cause epilepsy when disrupted in human infants [29]. The gene overlapping with ENSMUSG00000044407, ENSMUSG00000062078, encodes a protein involved in a number of processes including neuron myelination [30]. Both overlapping genes are plausible essential genes.

**Table 4:** Knockout experiments and SNPs within mouse *de novo* genes.

EnsEMBL ID	Overlapping genes	Knock-outs	SNPs
ENSMUSG00000075472	3′ UTR of ENSMUSG00000018363 on the same strand		1 NS in PWK/PhJ
ENSMUSG00000075582	1st intron and some coding sequence of ENSMUSG00000046352 on opposite strand		2NS total: 1NS in 2 strains, 1NS in WSB/EiJ
ENSMUSG00000037982	5′ UTR and 1st exon and intron of ENSMUSG00000038250 on opposite strand	knocked out in cell line, no phenotype in live mouse yet	5NS and 1S total: 4NS in Spretus/EiJ, 1NS and 1S in 14 strains
ENSMUSG00000078384	some coding and intronic sequence of ENSMUSG00000047730 on opposite strand		3NS and 1S total: 2NS and 1S in Spretus/EiJ, 1NS in 6 strains
ENSMUSG00000057354	intronic sequence of ENSMUSG00000054256 on opposite strand		4NS and 2S total: 2NS in 5 strains, 2S in Spretus/EiJ, 1NS in 2 strains, 1NS in 3 strains
ENSMUSG00000070700	some coding sequence of ENSMUSG00000050966 on opposite strand		3NS total: 2NS in Spretus/EiJ and 1NS in 2 strains
ENSMUSG00000074517*			5NS and 2S total: 3NS in PWK/PhJ (2 in same codon producing premature stop), 1S in Spretus/EiJ, 2NS in 2 strains, 1S in 6 strains
ENSMUSG00000073388		knocked out in cell line, no phenotype in live mouse yet	2NS and 3S total: 3S in Spretus/EiJ, 1NS in Spretus/EiJ, 1NS in CAST/EiJ
ENSMUSG00000075433*	some intronic and coding sequence of ENSMUSG00000022469 on opposite strand		8NS and 4S total: 1NS and 3S in CAST/EiJ, 4NS in 12 srains, 1NS in 11 strains (removing start codon), 1S in Spretus/EiJ, 1NS in 2 strains, 1NS in 10 strains
ENSMUSG00000043805			3NS in Spretus/EiJ
ENSMUSG00000048603	5′ UTR and 1st exon and intron of ENSMUSG00000021660 on opposite strand	knocked out in cell line, no phenotype in live mouse yet	7NS and 4S total: 1S and 5NS in Spretus/Eij, 1S in LPJ, 1S in CAST/EiJ, 1NS in 8 strains, 1S in 8 strains

* SNPs disrupt the valid ORF.

NS – nonsynonymous SNPs.

S – synonymous SNPs.

We could not identify any literature concerning the function of any of the knocked out *de novo* genes, so their functions remain unclear. We expect that the complete set of 75 murine genes we present will be of particular interest to researchers and single-gene knockout or knockdown studies should be performed on each one.

### Sequence conservation among mouse strains

We searched the mouse genome database, which contains sequence information for 17 mouse strains, for SNPs located within the coding sequences of the 11 best supported *de novo* candidates [31]. The coding sequences for each strain were aligned and translated. Generally speaking, these regions have low diversity. Only two of the 11 ORFs are disrupted in any strain (Table 4). In the case of gene ENSMUSG00000074517, two adjacent SNPs in one strain introduce a premature stop codon. For gene ENSMUSG00000075433, a SNP present in 11 strains removes its start codon, and therefore its coding potential. This polymorphism is identical to an enabler we identified as having been responsible for the creation of the ORF. According to the phylogeny of these mouse strains [32], the six strains containing the valid start codon do not form a clade to the exclusion of the other 11. Thus this may be an old polymorphism within mice that pre-dates the strain divergences.

## Discussion

We present strong evidence for the existence of a total of 75 murine *de novo* genes (Table 1; Table 2). Of these, 11 mouse cases have extremely strong support (Table 3): they are not found in any other lineages; there are no unannotated ORFs in the homologous regions in rat that could be orthologs; they all have transcription and peptide support; and their creation can be traced through some simple enabling mutations.

The fact that mouse and rat are each other's closest relatives and both display accelerated rates of evolution [20] means there is likely to be a large number of rearrangements in both species, and the problem is further compounded by their long divergence time. Additionally, the most closely-related outgroup species with adequate sequence data are guinea pig and human. The long evolutionary distances and the low sequence-coverage in guinea pig decrease the chances of discovering the orthologous DNA in the outgroups.

While mouse and rat have rapid rates of evolution when compared to other mammals, they have similar rates to each other. We would therefore expect the rate of *de novo* gene creation to be similar in the two species, yet we identified many more cases in mouse. However, our analysis began with fewer potential *de novo* cases in rat than in mouse (350 as opposed to 480). The difference may just be due to the relative incompleteness of the sequencing and annotation of the rat genome. An additional contributing factor may be the fact that orthology with mouse and human was used to some extent in the original annotation of the rat genome [33]. This may have resulted in the exclusion of some rat-specific genes.

**Figure 2:**
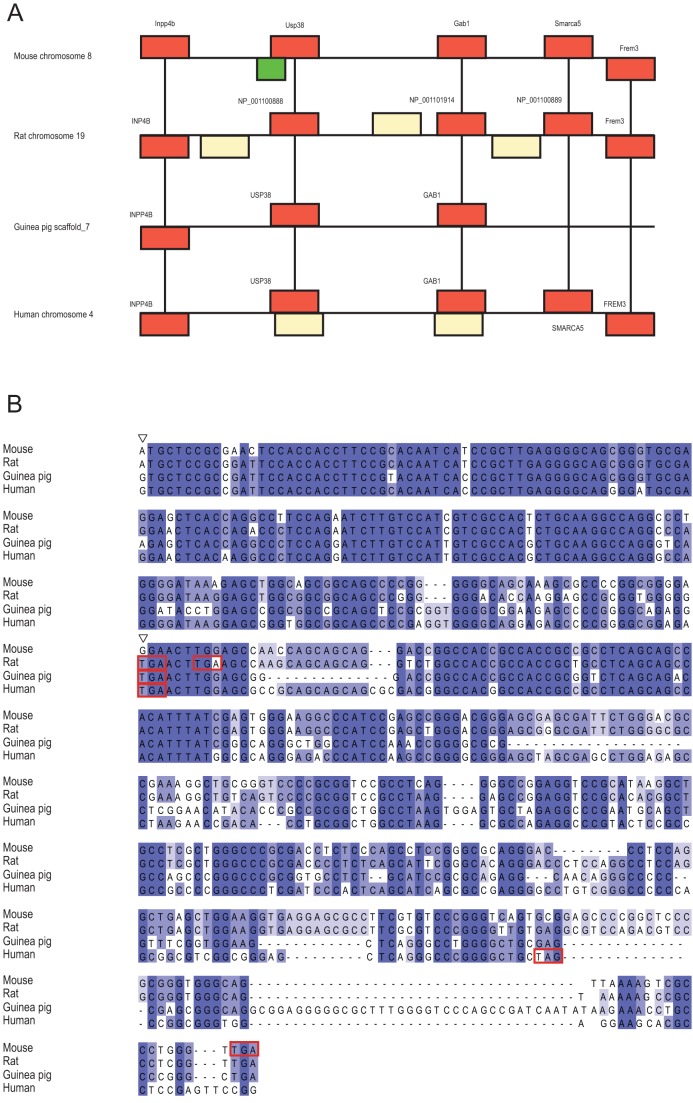
Ancestral regions of mouse gene ENSMUSG00000037982. A: Conserved synteny of the orthologous region containing the ancestral sequence of the gene in mouse, rat, guinea pig and human. Red boxes indicate orthologous genes, yellow boxes indicate non-orthologous genes, and the green box represents the location of the *de novo* gene. B: Alignment of the coding sequence of ENSMUSG00000037982 with the ancestral sequence present in rat, guinea pig and human. Red boxes indicate the locations of stop codons and empty triangles indicate the positions of the enabling mutations.

**Figure 3:**
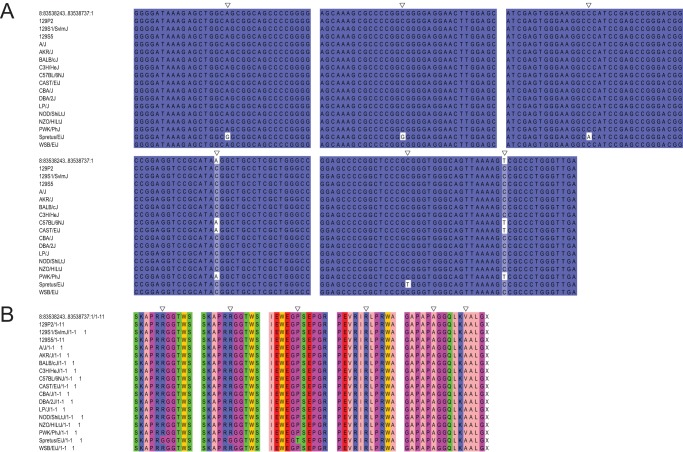
Alignment of the coding sequence of ENSMUSG00000037982 with 17 different mouse strains. In each alignment the mouse reference sequence taken from Ensembl is in the top row. 3A: Sections of the coding sequence available from Ensembl are aligned with the sequences for 17 different mouse strains taken from the Mouse Genome Project database. SNPs are indicated by empty triangles. 3B: Translated peptide sequences for each of the sections in 3A. The locations of each of the non-synonymous and synonymous SNPs are again indicated by empty triangles.

There are two mouse genes with particularly good support for their *de novo* origin, those with Ensembl identifiers ENSMUSG00000037982 and ENSMUSG00000078384. The gene with identifier ENSMUSG00000037982 is located on chromosome 8, opposite to *Usp38*, and encodes a protein 164 amino acids (aa) in length. The authenticity of this as a protein-coding gene is supported by 10 sequenced peptides, and mRNA evidence from multiple sources (Table 1). Two enablers seem to have been involved in its creation (Fig. 2). The first mutation is a G to A transition producing a start codon that is also present in rat, and therefore most likely occurred before the lineage divergence. Both guinea pig and human possess a G at this position and we infer this to be the same as the ancestral sequence. The second mutation is a mouse-specific G to T transversion removing a stop codon that is present in the other three species. One synonymous SNP and one nonsynonymous SNP are found in 14 mouse strains, and one strain contains four other SNPs (Fig. 3). Overall, the sequence conservation amongst mouse strains is high. The gene has been knocked out in a cell line, but so far there have been no reported experiments in a whole organism.

**Figure 4:**
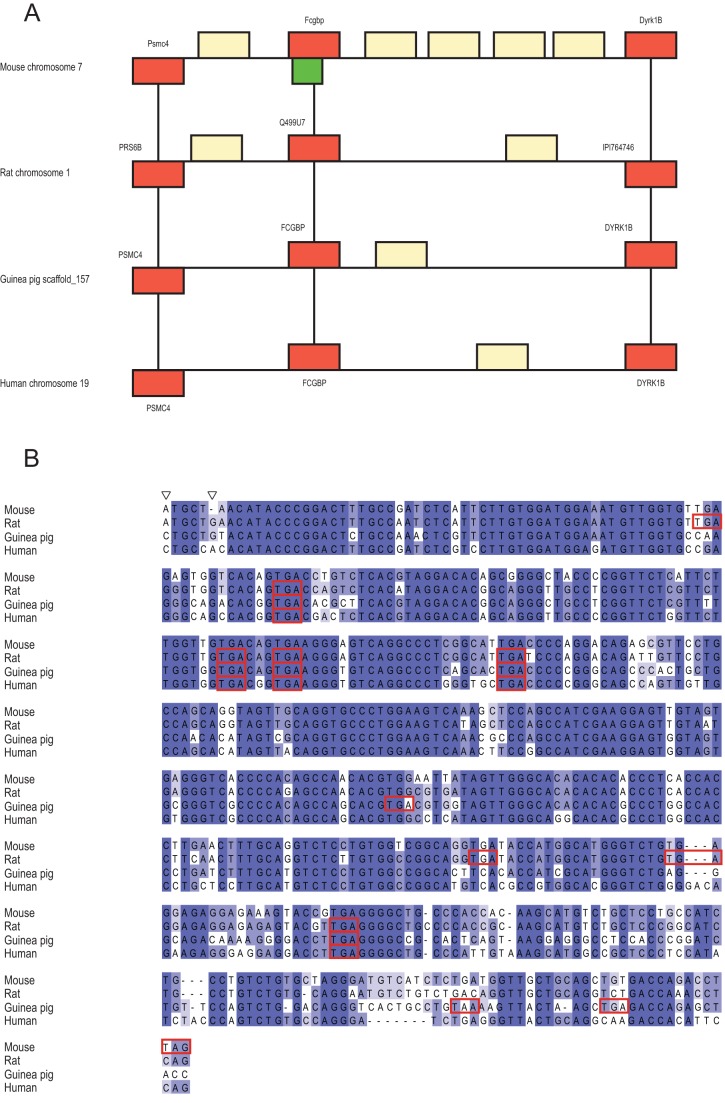
Ancestral regions of mouse gene ENSMUSG00000078384. 4A: Conserved synteny of the orthologous region containing the ancestral sequence of the gene in mouse, rat, guinea pig and human. Red boxes indicate orthologous genes, yellow boxes indicate non-orthologous genes, and the green box represents the location of the *de novo* gene. 4B: Alignment of the coding sequence of ENSMUSG00000078384 with the ancestral sequence present in rat, guinea pig and human. Red boxes indicate the locations of stop codons and empty triangles indicate the positions of the enabling mutations.

**Figure 5:**
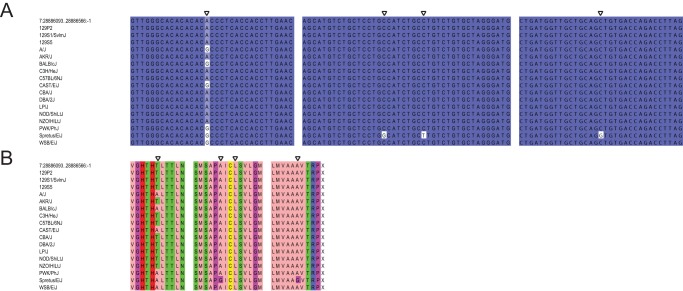
Alignment of the coding sequence of ENSMUSG00000078384 with 17 different mouse strains. In each alignment the mouse reference sequence taken from Ensembl is in the top row. 5A: Sections of the coding sequence available from Ensembl are aligned with the sequences for 17 different mouse strains taken from the Mouse Genome Project database. SNPs are indicated by empty triangles. 5B: Translated peptide sequences for each of the sections in 5A. The locations of each of the non-synonymous and synonymous SNPs are again indicated by empty triangles.

ENSMUSG00000078384 encodes a protein 157 aa in length and is located on chromosome 7, overlapping with, but on the opposing strand to, *Fcgbp* (Fig. 4A). Possibly as a result of functional constraints on the overlapping gene, sequence conservation is very high in this region across all four species (Fig. 4B). Two enablers seem to have been responsible for the birth of the mouse ORF. As with ENSMUSG00000037982, the first enabler occurred in the rat/mouse ancestor and resulted in the creation of a potential start codon, this time through a C to A transversion. The second enabler is a mouse-specific deletion of 1 base causing a frameshift, thus avoiding downstream stop codons. Sequence conservation is quite high across other strains (Fig. 5). Three SNPs are reported in one strain, and another SNP is found in a total of 6 strains.

### Characteristic features of *de novo* genes

All of the 11 strongest mouse candidates are small genes, and the predicted proteins are short, with lengths between 62 and 174 aa. The other 58 mouse genes for which we were unable to find unequivocal enablers have a similar range in size, from 40 to 184 aa, as do the 6 rat candidates (70 to 208 aa). In terms of peptide composition, not a single gene out of the entire 75 encodes a protein containing a known domain or functional motif, nor do they show any relatedness to other proteins. Examination of amino acid content also did not reveal any patterns. While many of the encoded peptides tend to show a high frequency of one residue or another, the particular residue varies from gene to gene. The lack of discernible patterns among the encoded peptides is not surprising considering the origin of the genes. It also indicates that there is no particular bias in *de novo* gene retention.

The coding sequences for each of the 11 mouse genes, and most of the other candidates, are contained within one exon. Of the entire set of 75 *de novo* candidates, only 5 mouse genes and 4 rat genes contain introns within their coding sequences. There were no introns in any of the 11 strongest mouse candidate genes. The presence of the introns is inferred from expression evidence, and their lengths range between 100 and 10,000 bases. In each case the intronic DNA is identifiable in the orthologous regions of other species, meaning they are unlikely to have appeared from insertions. Overall, the simple features of the candidate genes lend plausibility to their *de novo* origins (Knowles and McLysaght 2009).

Another common feature of *de novo* genes is, while their coding sequences are unrelated to existing protein-coding regions, they tend to be in the vicinity of, and often overlap with, other genes, either within introns, or on the opposite strand [13], [34]. 51 out of the 69 mouse genes overlap with other genes, 8 of which belong to the 11 strongest candidates. Of the six rat genes, three overlap with others (Table 2).

There are two possible explanations for these patterns. The first is that a simple structure, small size and close proximity to another gene may be required to facilitate the origin of a gene from non-coding DNA. In terms of their size and lack of introns, *de novo* genes, particularly young ones, are unlikely to evolve long ORFs and complex splicing signals simultaneously. Overlap with other genes provides a ready mechanism to enable transcription of the new genes. Thus, these frequently reported features in *de novo* genes may reflect common steps in their origins [35].

Another possibility, however, is that the common features are merely due to ascertainment biases resulting from the methods that are used to detect the *de novo* genes. We require relatively well-conserved synteny and identifiable and alignable homologous sequence between species in order to provide positive evidence of the absence of the gene from other lineages. Short genes that overlap with conserved genes are more likely to satisfy these criteria.

## Concluding Remarks

The origin of protein-coding genes *de novo* is increasingly recognized as a rare but consistent feature of eukaryotic genomes. As these genes are unique to particular species or clades, they could be responsible for some unique traits [19]. However, despite the wealth of data on mouse and rat in general, data on these genes of interest were sparse. Of the 75 cases that we report, not a single one contains a recognizable protein domain. This is not unexpected considering the nature of origin of these genes.

During the course of this study the Ensembl database was updated and a number of the mouse genes we present here were removed from the database (40 out of 69). The sequences in the corresponding regions remain unchanged in the most recent version of Ensembl (v66 at time of writing), and the expression and peptide evidence are still available for each gene. The genes were removed because of their lack of orthologs in other species, yet *de novo* genes, by their very definition, will not have any homologous genes in other species. It is therefore likely that the *de novo* origin of genes is more frequent than was initially thought, and many of them remain undiscovered. Robust identification of *de novo* genes will probably require more primary data such as RNA-seq as the starting point to infer the presence of genes.

As a result of the extremely strict criteria we used to define the mouse- and rat-specific *de novo* genes it is likely that the number of *de novo* genes present in each species is higher than what we have found. While the functions and the importance of each of the genes are not yet known, we have provided a list of extremely well-supported candidates for *de novo* gene origin which may be of interest for future functional analyses.

## Materials and Methods

### Sequence data

We obtained the complete set of 23497 mouse and 22938 rat protein-coding genes, along with their protein products from Ensembl v56 [36]. The initial set of *de novo* candidates in each of the two species were defined as protein-coding genes with no BLASTP hit in the other species with an expectation (e-) value better than 1×10^−3^. This resulted in a list of 350 rat and 480 genes.

### Search for homologous sequence

For each mouse and rat candidate novel gene the nucleotide sequence was used in a blastn search of the other species' genome. Only genes with a hit in the other genome at least 50% the length of the query gene, with a sequence identity of at least 70%, were kept in the data set. The numbers of potential *de novo* genes were reduced to 200 and 131 in mouse and rat, respectively.

### Removal of genes with orthologs in other species

Using the perl API, the Ensembl compara database was used to search for potential orthologs in other non-murine species. Any genes with a valid ortholog in another species were excluded from the dataset. Orthologs were only considered valid if they contained an ATG start codon and if each of their introns was at least 18 base pairs (bp) long. Short introns (1–5 bp) are often inferred by automated pipelines such as Ensembl in order to avoid frameshifts that would discount the presence of a gene, yet there is no evidence that introns shorter than 18 bases can be spliced [37]. It is possible that some mutations in these specific regions would have been responsible for the creation of *de novo* genes.

After excluding genes with blastp hits in other species 174 mouse genes and 95 rat genes remained.

### Removal of candidates with potential unannotated orthologs

Protein sequences for each of the potential *de novo* genes were used in a tblastn search of the appropriate genome. Regions of the genomes containing any hits with an e-value of 1×10^−3^ or better, along with 1000 bases of flanking sequence on either side, were taken as possible homologous sequence and were searched for any unannotated ORFs. Potential introns within these had to be at least 18 bp in length for the ORF to be considered valid. If the translated ORF aligned over at least 50% of the length of the candidate novel gene with at least 60% sequence identity then it was considered as a valid, unannotated ortholog.

### Other dataset refinements

We removed any *de novo* candidates lacking an ATG start codon, or containing any introns less than 18 bases in length. We were left with 152 potential *de novo* genes for mouse and 53 for rat.

### Expression and peptide evidence

We searched UniGene [38], which contains information from several different mRNA databases, for expression evidence for each of the *de novo* candidates.

The PeptideAtlas [25] and PRIDE [26] protein databases were searched for evidence of protein-coding potential for the *de novo* genes. Only peptides that uniquely matched the *de novo* gene under scrutiny were considered.

### Removal of candidates with potential GenBank orthologs

The protein sequences of each of the potential *de novo* genes were BLASTed against GenBank [39]. Any hits in other species with e-values lower than 1×10^−3^ covering at least 50% of the length of the gene were taken to be orthologs. This resulted in the exclusion of two rat genes.

### Enabling Mutations

The coding sequence for each of the 69 mouse genes was BLASTed against the entire human and guinea pig genomes. Hits with over 50% sequence identity and covering at least 50% of the gene were taken as possible homologous regions. Synteny was used wherever possible to confirm the homologous regions. As a result of the extensive divergence between mouse and the two outgroup species, the ancestral sequences proved to be difficult to determine, and were only found for 11 out of the 69 *de novo* candidates.

MUSCLE [27] was used to align the sequences of each of the *de novo* mouse genes with the homologous regions in rat, human and guinea pig. Alignments were then manually curated using Jalview [40], and were examined for lineage-specific mutations.

BLAST searches against the WGS trace data were performed using the NCBI BLAST website (www.ncbi.nlm.nih.gov/blast/) to obtain sequence traces for each of the regions containing the enabling mutations (Figures S1, S2, S3, S4, S5, S6, S7, S8, S9, S10, S11). Traces were only available for mouse, rat and human. They were examined in order to confirm there was no ambiguity with respect to the nucleotides present at the enabler locations.

### Peptide composition

Amino acid compositions for each of the proteins encoded by the *de novo* candidates were calculated using the ProtParam tool available on the ExPASy website (web.expasy.org/protparam/).

For each encoded protein, the PROSITE database was searched for peptide domains and motifs using the ScanProsite tool [41].

## Supporting Information

Figure S1 **Sequence traces for ENSMUSG00000073388.** A: Reverse complement of mouse sequence. B: Human sequence.
(EPS)

Figure S2 **Sequence traces for ENSMUSG00000075433.** A: Mouse sequence. B: Reverse complement of rat sequence. C: Reverse complement of human sequence.
(EPS)

Figure S3 **Sequence traces for ENSMUSG00000043805.** A: Mouse sequence. B: Reverse complement of rat sequence. C: Human sequence.
(EPS)

Figure S4 **Sequence traces for ENSMUSG00000048603.** A: Mouse sequence. B: Reverse complement of rat sequence.
(EPS)

Figure S5 **Sequence traces for ENSMUSG00000075472.** A: Mouse sequence. B: Rat sequence. C: Human sequence.
(EPS)

Figure S6 **Sequence traces for ENSMUSG00000075582.** A: Reverse complement mouse sequence. B: Rat sequence. C: Human sequence.
(EPS)

Figure S7 **Sequence traces for ENSMUSG00000037982.** A: Reverse complement of mouse sequence. B: Human sequence.
(EPS)

Figure S8 **Sequence traces for ENSMUSG00000078384.** A: Mouse sequence. B: Reverse complement of rat sequence. C: Human sequence.
(EPS)

Figure S9 **Sequence traces for ENSMUSG00000057354.** A: Mouse sequence. B: Human sequence.
(EPS)

Figure S10 **Sequence traces for ENSMUSG00000070700.** A: Mouse sequence. B: Rat sequence. C: Reverse complement of human sequence.
(EPS)

Figure S11 **Sequence traces for ENSMUSG00000074517.** A: Mouse sequence. B: Rat sequence. C: Reverse complement of human sequence.
(EPS)

## References

[1] Long M, Betran E, Thornton K, Wang W (2003) The origin of new genes: glimpses from the young and old. Nat Rev Genet 4: 865–875. 14634634 10.1038/nrg1204

[2] Chen L, DeVries AL, Cheng CH (1997) Convergent evolution of antifreeze glycoproteins in Antarctic notothenioid fish and Arctic cod. Proc Natl Acad Sci U S A 94: 3817–3822. 9108061 10.1073/pnas.94.8.3817 PMC20524

[3] Cai J, Zhao R, Jiang H, Wang W (2008) De novo origination of a new protein-coding gene in Saccharomyces cerevisiae. Genetics 179: 487–496. 18493065 10.1534/genetics.107.084491 PMC2390625

[4] Carvunis AR, Rolland T, Wapinski I, Calderwood MA, Yildirim MA, et al (2012) Proto-genes and de novo gene birth. Nature 487: 370–374. 22722833 10.1038/nature11184 PMC3401362

[5] Begun DJ, Lindfors HA, Kern AD, Jones CD (2007) Evidence for de novo evolution of testis-expressed genes in the Drosophila yakuba/Drosophila erecta clade. Genetics 176: 1131–1137. 17435230 10.1534/genetics.106.069245 PMC1894579

[6] Chen ST, Cheng HC, Barbash DA, Yang HP (2007) Evolution of hydra, a recently evolved testis-expressed gene with nine alternative first exons in Drosophila melanogaster. PLoS Genet 3: e107. 17616977 10.1371/journal.pgen.0030107 PMC1904467

[7] Levine MT, Jones CD, Kern AD, Lindfors HA, Begun DJ (2006) Novel genes derived from noncoding DNA in Drosophila melanogaster are frequently X-linked and exhibit testis-biased expression. Proc Natl Acad Sci U S A 103: 9935–9939. 16777968 10.1073/pnas.0509809103 PMC1502557

[8] Zhou Q, Zhang G, Zhang Y, Xu S, Zhao R, et al (2008) On the origin of new genes in Drosophila. Genome Res 18: 1446–1455. 18550802 10.1101/gr.076588.108 PMC2527705

[9] Zhang YE, Vibranovski MD, Krinsky BH, Long M (2010) Age-dependent chromosomal distribution of male-biased genes in Drosophila. Genome Res 20: 1526–1533. 20798392 10.1101/gr.107334.110 PMC2963816

[10] Chen S, Zhang YE, Long M (2010) New genes in Drosophila quickly become essential. Science 330: 1682–1685. 21164016 10.1126/science.1196380 PMC7211344

[11] Yang Z, Huang J (2011) De novo origin of new genes with introns in Plasmodium vivax. FEBS Lett 585: 641–644. 21241695 10.1016/j.febslet.2011.01.017

[12] Toll-Riera M, Bosch N, Bellora N, Castelo R, Armengol L, et al (2009) Origin of primate orphan genes: a comparative genomics approach. Mol Biol Evol 26: 603–612. 19064677 10.1093/molbev/msn281

[13] Knowles DG, McLysaght A (2009) Recent de novo origin of human protein-coding genes. Genome Res 19: 1752–1759. 19726446 10.1101/gr.095026.109 PMC2765279

[14] Li CY, Zhang Y, Wang Z, Cao C, Zhang PW, et al (2010) A human-specific de novo protein-coding gene associated with human brain functions. PLoS Comput Biol 6: e1000734. 20376170 10.1371/journal.pcbi.1000734 PMC2845654

[15] Wu DD, Irwin DM, Zhang YP (2011) De novo origin of human protein-coding genes. PLoS Genet 7: e1002379. 22102831 10.1371/journal.pgen.1002379 PMC3213175

[16] Zhang YE, Vibranovski MD, Landback P, Marais GA, Long M (2010) Chromosomal redistribution of male-biased genes in mammalian evolution with two bursts of gene gain on the X chromosome. PLoS Biol 8. 10.1371/journal.pbio.1000494 PMC2950125 20957185

[17] Xiao W, Liu H, Li Y, Li X, Xu C, et al (2009) A rice gene of de novo origin negatively regulates pathogen-induced defense response. PLoS One 4: e4603. 19240804 10.1371/journal.pone.0004603 PMC2643483

[18] Heinen TJ, Staubach F, Haming D, Tautz D (2009) Emergence of a new gene from an intergenic region. Curr Biol 19: 1527–1531. 19733073 10.1016/j.cub.2009.07.049

[19] Khalturin K, Hemmrich G, Fraune S, Augustin R, Bosch TC (2009) More than just orphans: are taxonomically-restricted genes important in evolution? Trends Genet 25: 404–413. 19716618 10.1016/j.tig.2009.07.006

[20] Adkins RM, Gelke EL, Rowe D, Honeycutt RL (2001) Molecular phylogeny and divergence time estimates for major rodent groups: evidence from multiple genes. Mol Biol Evol 18: 777–791. 11319262 10.1093/oxfordjournals.molbev.a003860

[21] Jacobs LL, Pilbeam D (1980) Of mice and men: fossil-based divergence dates and molecular “clocks.”. J Hum Evol 9: 551–555.

[22] Kumar S, Hedges SB (1998) A molecular timescale for vertebrate evolution. Nature 392: 917–920. 9582070 10.1038/31927

[23] Wilson AC, Carlson SS, White TJ (1977) Biochemical evolution. Annu Rev Biochem 46: 573–639. 409339 10.1146/annurev.bi.46.070177.003041

[24] Birney E, Andrews TD, Bevan P, Caccamo M, Chen Y, et al (2004) An overview of Ensembl. Genome Res 14: 925–928. 15078858 10.1101/gr.1860604 PMC479121

[25] Deutsch EW, Lam H, Aebersold R (2008) PeptideAtlas: a resource for target selection for emerging targeted proteomics workflows. EMBO Rep 9: 429–434. 18451766 10.1038/embor.2008.56 PMC2373374

[26] Vizcaino JA, Cote R, Reisinger F, Foster JM, Mueller M, et al (2009) A guide to the Proteomics Identifications Database proteomics data repository. Proteomics 9: 4276–4283. 19662629 10.1002/pmic.200900402 PMC2970915

[27] Edgar RC (2004) MUSCLE: multiple sequence alignment with high accuracy and high throughput. Nucleic Acids Res 32: 1792–1797. 15034147 10.1093/nar/gkh340 PMC390337

[28] Skarnes WC, Rosen B, West AP, Koutsourakis M, Bushell W, et al (2011) A conditional knockout resource for the genome-wide study of mouse gene function. Nature 474: 337–342. 21677750 10.1038/nature10163 PMC3572410

[29] Marshall CR, Young EJ, Pani AM, Freckmann ML, Lacassie Y, et al (2008) Infantile spasms is associated with deletion of the MAGI2 gene on chromosome 7q11.23–q21.11. Am J Hum Genet 83: 106–111. 18565486 10.1016/j.ajhg.2008.06.001 PMC2443840

[30] Sidman RL, Dickie MM, Appel SH (1964) Mutant Mice (Quaking and Jimpy) with Deficient Myelination in the Central Nervous System. Science 144: 309–311. 14169723 10.1126/science.144.3616.309

[31] Gregory SG, Sekhon M, Schein J, Zhao S, Osoegawa K, et al (2002) A physical map of the mouse genome. Nature 418: 743–750. 12181558 10.1038/nature00957

[32] Beck JA, Lloyd S, Hafezparast M, Lennon-Pierce M, Eppig JT, et al (2000) Genealogies of mouse inbred strains. Nat Genet 24: 23–25. 10615122 10.1038/71641

[33] Gibbs RA, Weinstock GM, Metzker ML, Muzny DM, Sodergren EJ, et al (2004) Genome sequence of the Brown Norway rat yields insights into mammalian evolution. Nature 428: 493–521. 15057822 10.1038/nature02426

[34] Makalowska I, Lin CF, Hernandez K (2007) Birth and death of gene overlaps in vertebrates. BMC Evol Biol 7: 193. 17939861 10.1186/1471-2148-7-193 PMC2151771

[35] Siepel A (2009) Darwinian alchemy: Human genes from noncoding DNA. Genome Res 19: 1693–1695. 19797681 10.1101/gr.098376.109 PMC2765273

[36] Hubbard TJ, Aken BL, Beal K, Ballester B, Caccamo M, et al (2007) Ensembl 2007. Nucleic Acids Res 35: D610–617. 17148474 10.1093/nar/gkl996 PMC1761443

[37] Gilson PR, McFadden GI (1996) The miniaturized nuclear genome of eukaryotic endosymbiont contains genes that overlap, genes that are cotranscribed, and the smallest known spliceosomal introns. Proc Natl Acad Sci U S A 93: 7737–7742. 8755545 10.1073/pnas.93.15.7737 PMC38817

[38] Wheeler DL, Church DM, Federhen S, Lash AE, Madden TL, et al (2003) Database resources of the National Center for Biotechnology. Nucleic Acids Res 31: 28–33. 12519941 10.1093/nar/gkg033 PMC165480

[39] Benson DA, Karsch-Mizrachi I, Lipman DJ, Ostell J, Sayers EW (2011) GenBank. Nucleic Acids Res 39: D32–37. 21071399 10.1093/nar/gkq1079 PMC3013681

[40] Waterhouse AM, Procter JB, Martin DM, Clamp M, Barton GJ (2009) Jalview Version 2– a multiple sequence alignment editor and analysis workbench. Bioinformatics 25: 1189–1191. 19151095 10.1093/bioinformatics/btp033 PMC2672624

[41] de Castro E, Sigrist CJ, Gattiker A, Bulliard V, Langendijk-Genevaux PS, et al (2006) ScanProsite: detection of PROSITE signature matches and ProRule-associated functional and structural residues in proteins. Nucleic Acids Res 34: W362–365. 16845026 10.1093/nar/gkl124 PMC1538847

